# A Glimpse of Things to Come: Featured Abstracts from the 18th National Conference on Chronic Disease Prevention and Control

**Published:** 2004-03-15

**Authors:** David L. Katz

**Affiliations:** Yale-Griffin Prevention Research Center

Great ventures are invariably a blend of tradition and innovation. Tradition is a cornerstone of identity, and without it, there is no clearly defined role, no unique character, no reliability. But in a constantly changing world, tradition without innovation is a cumbrous burden. Innovation allows a great venture to keep pace with the currents of change, to remain great and timely.

The annual Chronic Disease Conference is such a venture. Spanning 18 years, the conference has given rise to an array of traditions. It is tradition for the conference to address timely, compelling public health issues. It is tradition for the conference to encompass the breadth and depth of matters relating to preventing chronic disease. It is tradition for content to be shaped by the thoughtful reflection of invited luminaries and the insightful advances conveyed within abstracts submitted for competitive review.

Conference traditions are now to be enhanced by a well-timed innovation: the launch of *Preventing Chronic Disease: Public Health Research, Practice, and Policy* has created a unique capacity to publish select conference abstracts concurrently with the conference. The efficiencies of electronic publication allow for identifying, selecting, and editing these submissions as the conference itself undergoes final preparation. The 21 most meritorious abstracts from this year's conference appear within this issue of *Preventing Chronic Disease*. This innovation will itself count among the traditions that define the character, content, and excellence of the Chronic Disease Conference for years to come.

Advantages of this effort are considerable. E-journal readers unable to attend the February conference may nonetheless gain an appreciation for the work of their colleagues. Virtual networking will enhance conference networking, and individuals not accustomed to attending the meeting may be enticed to do so.

Publication is the ultimate "carrot and stick" of academic advancement. Those among us who must be calculating in our use of time now have an added incentive to submit our best work to the Chronic Disease Conference for consideration. The caliber of abstracts the conference planners are privileged to review has always been excellent, and the promise of publication is motivation to ever greater excellence.

With topics ranging from nutrition in schoolchildren to stroke in the elderly, from secondhand smoke to homelessness, the abstracts that follow provide a window to the dynamic work, rigorous science, dedicated effort, and passion for public welfare that so richly furnish the Chronic Disease Conference. They provide, as well, a glimpse of things to come. *Preventing Chronic Disease* will from now on reward the most laudable of our submissions with online publication. The content of the abstracts directs our efforts and imaginations to the best of innovations in public health practice and to advances in our collective capacity to prevent chronic disease.

